# 小细胞肺癌全程随访管理模式的探索与挑战

**DOI:** 10.3779/j.issn.1009-3419.2025.106.01

**Published:** 2025-01-20

**Authors:** Chengming HUANG, Yongzhao ZHOU, Jing XU, Wenting LU, Li TU, Yalun LI, Panwen TIAN

**Affiliations:** ^1^610041 成都，四川大学华西医院全程管理中心; ^1^Integrated Care Management Center, West China Hospital, Sichuan University, Chengdu 610041, China; ^2^610041 成都，四川大学华西医院肺癌中心; ^2^Lung Cancer Center, West China Hospital, Sichuan University, Chengdu 610041, China; ^3^610041 成都，四川大学华西医院呼吸与危重症医学科; ^3^Department of Respiratory and Critical Care Medicine, West China Hospital, Sichuan University, Chengdu 610041, China

**Keywords:** 肺肿瘤, 小细胞肺癌, 全程管理, 探索与挑战, Lung neoplasms, Small cell lung cancer, Whole course management, Exploration and challenge

## Abstract

小细胞肺癌（small cell lung cancer, SCLC）恶性程度高，其治疗模式及病程管理受到关注。熟悉SCLC的临床特点，掌握SCLC的筛查、诊断和治疗方法，及时处理治疗的不良反应，是制定科学合理的SCLC全程管理方案的基础。利用智能化全程随访管理平台，动态随访、及时预警、早期干预，使高质量的全生命周期管理成为可能。本文旨在对SCLC的治疗现状、全程管理模式的探索和挑战进行综述，为更好地管理SCLC患者、提高患者生存质量、改善患者的预后提供借鉴。

## Abstract (en)

Small cell lung cancer (SCLC) is a highly malignant disease that has garnered significant attention in terms of treatment modalities and course management. Gaining an understanding of the clinical characteristics of SCLC, acquiring proficiency in screening, diagnosis, and treatment methods for this condition, as well as promptly addressing any adverse reactions to treatment are essential foundations for developing a scientific and rational pathological management plan for SCLC. By utilizing an intelligent whole course follow-up management platform, dynamic follow-up, timely warnings, and early interventions can enable high-quality whole life cycle management. This article aims to review the current treatment landscape of SCLC while exploring the challenges associated with implementing a comprehensive process-oriented management approach. The goal is to provide valuable insights for better managing SCLC patients and ultimately improving their quality of life and prognosis.

肺癌是我国发病率和死亡率居首位的恶性肿瘤^[1]^，而小细胞肺癌（small cell lung cancer, SCLC）是肺癌中恶性程度最高、预后极差的肺恶性肿瘤^[2]^。SCLC在确诊时大部分已经处于广泛期，且具有治疗后容易复发、易转移、疗效差等特点^[3]^。如何早期发现、早诊断、及时就诊，已经成为临床SCLC诊疗策略中值得关注并亟待解决的问题。近年来，随着慢性病患者基数不断增大和人们生活习惯的转变，SCLC患者人数较以往也有不断增加的趋势^[4,5]^。SCLC患者对医疗资源需求度高，目前SCLC缺乏精准的治疗手段，对恶性肿瘤患者推行全程管理成为热点。全程管理是以患者为中心，包括疾病预防、疾病筛查、疾病诊治、康复随访、健康宣教等医护一体化的全程健康管理模式^[6,7]^。与传统就诊模式相比（图1），全程管理强调不同就诊阶段的连续性，解决患者就诊中频繁往返医院、治疗中各项复杂流程难沟通以及诊后无人管理的问题。智能化随访助力全程管理模式的开展，智能化随访是指基于互联网等主流技术，以专业的随访知识库为基础，提供医患沟通，实现院前、院中、院后随访和以健康宣教为核心的服务平台，通过智能化随访系统和多个人工智能工具的应用，提高医疗质量和随访效率，提升患者满意度和依从性，更好地实现全生命周期的随访管理^[8,9]^。将有限的医疗资源合理整合，全生命周期随访，打造离院不离医的模式，可以为探索SCLC的临床诊治、提高患者生存质量、延长生存时间提供新管理模式。

**图1:**
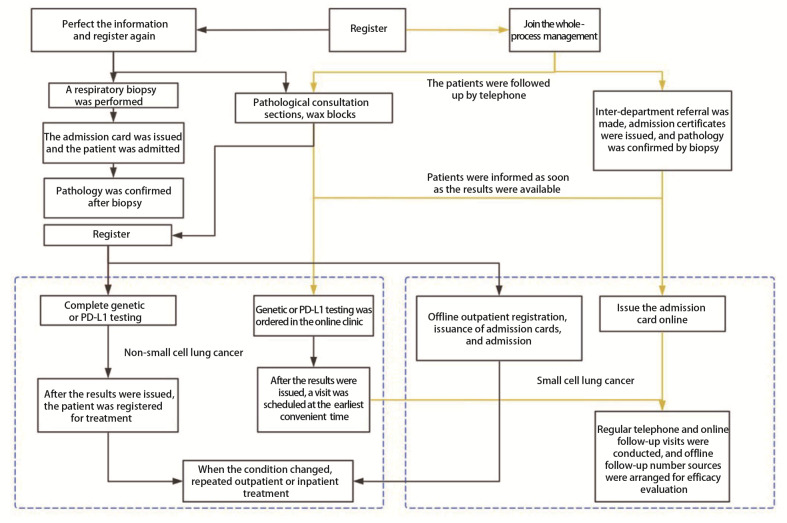
传统就诊流程与全程管理模式对比

## 1 SCLC的特点

SCLC是肺癌类型中的一种，约占所有肺癌的15%^[10]^，初始用药敏感但很快出现耐药，容易出现局部复发或/和远处转移，5年生存率低，约为5%^[11]^，患者在确诊为SCLC后需要长期反复住院和门诊就诊，在治疗中患者往往需要经历多线治疗，开发新的疗法难度大，改善SCLC治疗格局和预后是大家关注的焦点。

## 2 SCLC治疗策略的研究现状

依照美国退伍军人肺癌协会二期分期法^[12]^，SCLC主要分为局限期SCLC（limited stage SCLC, LS-SCLC）和广泛期SCLC（extensive stage SCLC, ES-SCLC），大部分患者在确诊的时候已出现转移，尽管在过去几十年里医疗技术不断取得进步，但是SCLC总的治疗策略进展缓慢，成效有限^[13]^。

LS-SCLC的治疗原则是以同步放化疗为主的综合治疗。有临床指南^[14]^及研究^[15⇓-17]^ 指出，cT1-2N0M0 SCLC的治疗可考虑手术，在Doerr等^[18]^的回顾性研究中对I和II期手术患者的平均生存期进行了*meta*分析，结果显示这两个阶段的患者可通过手术获得显著的生存期，I和II期SCLC患者应该考虑手术治疗，但LS-SCLC患者术后的临床疗效还缺乏前瞻性研究来验证^[19]^。在Cheng等^[20]^的研究中，评估对于接受标准铂类同步放化疗后未发生疾病进展的LS-SCLC肺癌患者，度伐鲁单抗（Durvalumab）进行巩固治疗，研究结果显示度伐鲁单抗治疗组的总生存期（overall survival, OS）显著长于安慰剂组[（55.9 *vs* 33.4个月，风险比（hazard ratio, HR）为0.73，*P*=0.01]，无进展生存期（progression‐free survival, PFS）也长于安慰剂组（16.6 *vs* 9.2个月，HR=0.76，*P*=0.02），该研究提供的数据显示度伐鲁单抗在LS-SCLC患者巩固治疗中的潜力很大。

ES-SCLC的治疗原则是以化疗为主的药物治疗，传统治疗方式未明显改善ES-SCLC患者生存期。ASTRUM-005研究^[21]^是一项既往未接受治疗的ES-SCLC患者对比斯鲁利单抗（Serplulimab）联合化疗与安慰剂联合化疗的临床研究，结果显示斯鲁利单抗组的中位OS为15.4个月（95%CI: 13.3-NE），明显超过安慰剂组的10.9个月（95%CI: 10.0-14.3），HR为0.63（95%CI: 0.49-0.82, *P*<0.001），斯鲁利单抗组的中位PFS为5.7个月（95%CI: 5.5-6.9），也比安慰剂组的4.3个月（95%CI: 4.2-4.5）更长，HR为0.48（95%CI: 0.38-0.59）。另有类似的IMpower133研究^[22⇓-24]^和CASPIAN研究^[25,26]^结果显示免疫治疗为ES-SCLC患者延长生存时间带来了希望之光。

免疫治疗可改善SCLC患者的预后，但仅有部分患者对免疫治疗有疗效反应，仍需要大量的临床研究开发新的治疗方法和预测标志物，如抗血管治疗、抗体药物偶联物（antibody-drug conjugates, ADC）类的药物、嵌合抗原受体T细胞疗法（chimeric antigen receptor T-cell therapy, CAR-T）等，为改善SCLC患者预后提供潜在手段。

## 3 SCLC全程管理策略研究

### 3.1 SCLC进行全程管理的重要性

习近平总书记在党的二十大报告中提出“加强重大慢性病健康管理”，随后政府部门相继出台的相关政策中提到人工智能、智慧医院、诊后随访，逐步推行慢病管理，结合我国实际情况，肿瘤患者基数大，发病率高，而医疗资源有限。改善患者的就医体验，加强诊后管理与随访，为患者提供专业的院外康复及延续性治疗，有效提升慢病患者的诊后体验，实现全程化、精细化的健康随访管理，这些成为了亟待解决的问题。而在慢病患者中肺癌位居我国恶性肿瘤之首，在国家政策引导下全程管理成为肺癌防治的趋势^[27]^。

SCLC治疗方案多年未得到提升和突破，是临床医生的难点、患者的痛点，对SCLC进行全程管理具有至关重要的意义。在接诊后治疗前，依靠影像学进行准确的分期，针对不同的分期选择合适的治疗方案可提高患者治疗效果；在诊治中，通过影像学和肿瘤标志物的评估，观察是否耐药，及时进行线下门诊就诊以便于调整用药方案；患者线上复诊时，通过自我体征的描述和血液检查，可发现并处理抗肿瘤治疗后的副作用；智能化随访能定期提醒患者复查复诊，有助于发现并处理复发或转移的情况。病情疑难需要多学科会诊时，可提供共病转诊通道，解决科室间不同医生挂号难问题，确保治疗的及时性，故在SCLC的治疗过程中，应重视并加强全程管理实施。

### 3.2 SCLC的预防和健康教育

众所周知，SCLC与吸烟关系密切，Huang 等^[28]^的研究利用*meta*分析，共纳入27项研究，包括12,047例SCLC患者（9137例吸烟者和2910例从不吸烟者），采用HR及其95%CI评估治疗前吸烟状态与患者生存的关系，使用RevMan 5.3和STATA 15.0软件进行统计学分析。结果显示，吸烟史与较差的生存结局密切相关（OS: HR=1.17, 95%CI: 1.12-1.23, *P*<0.00001, *I^2^*=0%; PFS: HR=1.20, 95%CI: 1.06-1.35, *P*=0.004, *I^2^*=0%），吸烟史是影响SCLC患者预后的独立不良因素。相似的文献^[29⇓-31]^中吸烟是引起SCLC的关键性因素，阻止吸烟和戒烟，早期发现病变，是降低发病率和死亡率的关键。利用智能化全程管理平台将戒烟、预防SCLC发生的健康宣教资料多途径推送给患者及家属，使其知道吸烟的危害，预防SCLC发生。患者在院就诊期间，肺癌专病管理师在全程随访中充分了解患者需求，总结患者共性问题，协同临床专家、专病推广师运用多媒体产出健康教育视频、图文等形成精准化宣教库，根据患者的具体病情，推送精准化教育内容至患者手机，并且通过病友会、专题讲座等形式加强与患者的沟通，提高患者对SCLC的认识水平，减缓其对疾病认识不足引起的焦虑^[9]^。

早期筛查是降低肺癌死亡率的有效且重要的方式，在刘丹等^[32]^的文献中构建肺结节/肺癌全程管理平台，将肺结节的早期筛查纳入肺癌的规范诊疗体系，提升肺癌患者的生存率，利用这一平台对患者进行早期规范诊疗，以“主动、全程、规范”为核心，通过规范筛查、规范随访、规范诊疗，实现肺癌早诊早治，降低肺癌患者的死亡率。此模式也有利于及时发现问题、及时处理，明确早期干预的方式，降低SCLC发现时即为晚期的风险，从预防角度提升SCLC患者的生存率，该模式为关注SCLC病情演变的关键靶点提供线索，达到降低死亡率的最终目标。

### 3.3 SCLC的筛查和诊断

SCLC生长速度快、易转移，早期筛查和诊断尤为重要。有研究^[33,34]^ 表明早期筛查、及时诊断并接受治疗可以有效改善SCLC的预后。在日常生活中对于长期吸烟或接触二手烟的人群（年龄50-80岁，烟龄至少20包/年），建议定期行胸部低剂量计算机断层扫描（low-dose computed tomography, LDCT）。Yang等的^[35]^研究提示与常规筛查相比，LDCT的筛查使早期肺癌的检出率提高74.1%。目前SCLC的常规筛查还包括血液肿瘤标志物的检测，有研究^[36,37]^报道SCLC常规的肿瘤标志物主要有神经元特异性烯醇化酶、细胞角蛋白片段、胃泌素释放肽前体，这些标志物可以辅助区分肺癌的组织学类型，为SCLC的筛查和诊断提供有价值的线索。早期通过LDCT扫描发现肺部肿瘤位置、大小以及淋巴结有无转移情况、进行病理活检是诊断SCLC的金标准，临床上获取病理组织的方法主要有经皮肺穿刺活检、支气管镜下活检、纵隔镜下和胸腔镜下活检。病理活检如提示SCLC，应立即开始抗肿瘤治疗，早期的筛查和诊断对于改善SCLC患者的生活质量及预后至关重要。

### 3.4 SCLC抗肿瘤治疗后副作用的管理

SCLC病情复杂，治疗手段有限，在短时间内需要多线治疗或者同时实行多种治疗方式，患者在接受诊治中存在不同程度的毒副反应，所涉及的副作用包括血液系统、消化系统、心血管系统、神经系统等多个系统，对身体损伤大，而肿瘤治疗相关的毒副作用是动态变化的。Cheng等^[20]^的研究数据显示，接受度伐鲁单抗治疗的患者，最高级别为3-4级不良反应的发生率为24.4%，不良反应导致停药的患者比例为16.4%，导致死亡的患者比例为2.7%，最高级别为3-4级的肺炎或放射性肺炎占3.1%。在CheckMate 032研究^[38]^中分析纳武利尤单抗（Nivolumab）单药治疗SCLC的不良反应，研究结果显示，接受纳武利尤单抗治疗后约有55%的患者发生了不良反应，发生3-4级不良反应的患者占11.9%，此研究报道了多数的不良反应事件，涉及多个身体系统，包括皮肤反应（21.1%）、内分泌指标异常（9.2%）、胃肠道反应（6.4%）、肝脏反应（4.6%）、肺部反应（1.8%）、肾脏反应（0.9%）等。类似的研究如KEYNOTE-028研究^[39]^、KEYNOTE-604研究^[40]^等都有不同程度毒副反应的报道。这些副作用不容忽视，对患者的生活质量产生负面影响，严重时会影响正常治疗时间，甚至威胁生命，因此在治疗前对患者病情进行全面评估、加强诊后随访具有重要的意义。面对个体差异性、疾病复杂程度，各抗肿瘤治疗科室如放疗科、介入科等之间应加强学科合作，以智能化全程随访管理平台为依托，为患者便利就诊提供转诊通道，可有效减少或者预防肿瘤治疗引起的并发症，及时处理毒副作用，减轻患者痛苦；而线上复诊，对患者疑问进行专业解答，及时和团队医生保持沟通，可减轻SCLC患者焦虑度，提高其生存质量，改善其预后。

### 3.5 SCLC全程管理的探索与实践

为践行国家政策，保障慢病患者规范化管理，四川大学华西医院在工作岗位中探索设置肺癌专病管理师对肺癌患者实施全程管理^[41]^，通过构建患者管理信息平台、设置肺癌专病管理师岗位职责、实施院前准备、住院管理、出院后随访等全程化闭环管理措施（肺癌患者全程管理模式）后，共管理患者5576例（在5576例患者中行纤维支气管镜活检患者186例，其中恶性肿瘤171例、良性肿瘤15例）；对比实施肺癌全程管理前1年内的190例活检患者（恶性肿瘤67例、良性肿瘤123例），实施管理后的患者活检筛查恶性比例（91.9%）高于实施前（35.3%），差异有统计学意义（*χ*^2^=129.939, *P*<0.001），提示肺癌专病管理师岗位的设置能够对可疑肺癌患者进行精准筛查，及时追踪患者病情，防止延误或遗漏患者诊断，提高了肺癌复发患者的诊疗质量，同时也提高了肺癌患者随访依从性和诊疗完成率。在陶文娟等^[27]^的研究中，通过患者导航模式提供个性化、连续性服务，解决肺癌患者全程管理中跨层级跨机构服务碎片化的困境，可为病情复杂肺癌患者匹配医疗资源，实现医联体背景下的全程管理。四川大学华西医院运用互联网平台和人工智能工具，实现肺癌高危人群队列筛选，规范肺癌诊疗及共病管理，实行全生命周期管理流程；全程管理成员协助临床团队，通过不同临床科室的转诊，对患者生活质量、心理、营养、疼痛等进行综合管理^[9]^；利用人工智能辅助诊断系统对患者进行辅助诊断，患者居家可自主完成线上随访评估，医护实时查看随访评估结果，如有异常系统自动预警，医护人员及时联系患者协助处理，保障患者在家能实时监测身体情况，并有团队进行管理，这为肺癌患者的全程管理提供了平台和高效的诊疗团队模式，保障了患者离院不离医的体验。肺癌全程管理实施框架见图2。

**图2:**
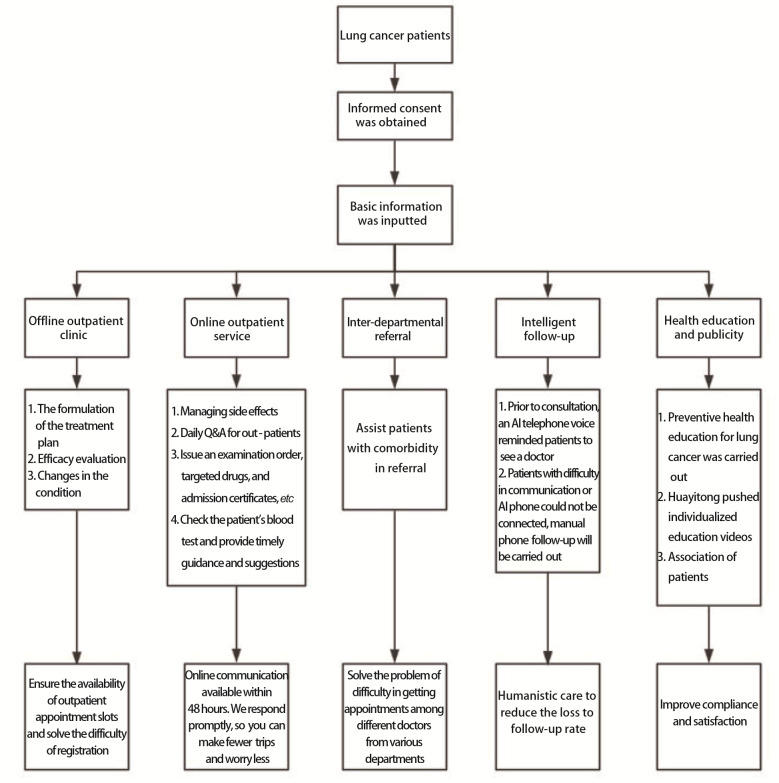
肺癌全程管理实施框架图

肺癌全程管理模式为SCLC患者指明了方向，目前还没有统一的随访管理标准，但SCLC是肺癌中恶性程度高、病情变化快、预后极差的一种类型，需要早期干预，规范化诊疗，实时监测病情，及时处理不良反应。王聪等^[41]^研究中提到，通过设置肺癌专病管理师，建立患者全程管理中心，对肺癌患者实行全程管理模式，实现了肺癌诊疗规范化管理。虽然研究中没有SCLC患者精准全程管理的数据支撑，但该模式为SCLC患者的管理提供了全新而高效的思路。结合SCLC的临床特点以及肺癌全程管理模式的搭建，可从早期进行疾病评估和诊断、个性化治疗方案的制定，诊治过程中对疗效进行检测、副作用的处理以及患者焦虑的心理疏导，使患者树立战胜疾病的信心；在患者出院后给予饮食、运动等方面的康复指导，定期随访观察，调整患者生活方式，可多维度地提高患者的疗效和生存质量。SCLC的全程管理在实践中将会面临更多的挑战，应通过加强基础研究、多学科团队诊疗，提高患者管理和随访频率，克服困难，进一步探索SCLC个体化全程管理新模式。

### 3.6 SCLC全程管理的困难与挑战

SCLC全程管理的困难与挑战是肿瘤学领域中备受关注的热点问题，下面从SCLC的特征、当前治疗管理的局限性及全程管理中面临的挑战进行分析。

SCLC是一种恶性程度高的肺癌类型，侵袭性强、分化程度低、易转移，治疗难度大。同时，虽然SCLC对化疗和放疗敏感，但易产生耐药，且复发率高，这些疾病特点是全程管理面临的困难。目前SCLC的治疗效果有限，且易产生副作用，尽管近年来免疫治疗等新型治疗方法为SCLC患者带来了新的希望，但单药免疫治疗在SCLC中的二线治疗地位尚未确立，且疗效依赖于肿瘤免疫微环境^[13]^，部分患者疗效不理想。SCLC患者的个体化差异较大，对于治疗的反应也不同，个体化治疗方案对于提高治疗效果至关重要，由于患者需要经历多线治疗，使得实施个体化全程管理难度较大。另外，SCLC的复发和转移是临床治疗的难点，大部分LS-SCLC和ES-SCLC都会复发且易转移至脑、肝、骨等关键器官，导致治疗难度加大，这也是全程管理中的重要难题。

SCLC患者病情进展迅速，需要密切监测和及时调整治疗方案，由于患者个体差异和疾病的复杂性，部分患者的依从性差，增加了患者的管理难度。SCLC患者的全程管理需要多学科协作，包括肿瘤内科、放疗科、胸外科、呼吸科等多个科室的共同参与，医疗资源的匹配、服务管理的连续性、信息反馈的及时性、治疗中需要消耗的大量医疗资源（如人力、药物以及设备）等，都对SCLC患者的全程管理提出严峻的考验。

面临上述诸多的困难和挑战，需要探索新型的治疗方法在SCLC中的应用，以提高治疗效果，延长生存期；需要加强个体化治疗，深入了解SCLC患者的个体差异和基因特征，制定个体化治疗方案；需要优化多学科协助模式，完善多学科协作机制，保证治疗方案的制定和实施更科学；需要提高医疗资源利用率，提高医疗技术和服务水平，为患者提供更优质、高效的医疗服务。

虽然SCLC全程管理面临诸多困难和挑战，但随着信息技术的发展，人类已经进入大数据时代，大数据和人工智能将会成为医疗行业发展的重要方向^[42]^。我国恶性肿瘤人口数量日趋升高，其中肺癌发病率和死亡率居首位^[43]^，肺癌的诊断或诊治中亟需引进新技术。目前利用大数据可化解临床数据结构多样性、质量不稳定等问题，在陈嘉旖等^[44]^研究中，上海市肺科医院在长期的临床数据基础上建立了肺癌专病数据库，医院利用该平台在3个月内处理了5年以上的临床数据信息，生成14万份肺癌专病病历数据，提高和巩固了医院在肺科疾病方面的科研领先地位。Kamran等^[45]^研究回顾性分析了诊断为LS-SCLC并接受放化疗治疗的105例患者，比较了基于CT定量肿瘤体积测量与肿瘤原发灶-淋巴结-转移（tumor-node-metastasis, TNM）分期来预测2年OS、局部复发和远处转移的结果，结果显示影像组学中肿瘤的延伸度与局部复发（HR=1.10, *P*=0.003）和2年OS（HR=1.10, *P*=0.03）有关，可能比TNM分期能更好地预测预后，提示人工智能对不同分期患者的生存预后的预测具有良好的效能，具有辅助获取临床预后信息方面的潜能。

医学领域大数据的日益完善与人工智能的不断发展对SCLC精准诊疗具有巨大的作用。根据患者的检查数据大数据做好分析判断，人工智能协助医生确定病因和进行方案的制定，并建立SCLC患者全程管理，不仅可以更好地观察和诊治患者，还可以全程了解病情变化趋势，从而改善SCLC的防治效果，为患者带来更好的生存质量和预后。

## 4 总结

SCLC的全程管理是一个复杂而细致的过程，涉及多个环节和多种治疗方式，涵盖了预防宣传、早期筛查和诊断、治疗中不良反应的及时处理、诊后康复等各个阶段的随访管理。SCLC的全程管理可扩大预防SCLC的宣教工作，增进对SCLC的认知，开展健康教育活动，加强患者间的交流与鼓励，同时也为家属提供必要的支持和指导。根据患者的身体情况、疾病分期、分子特征等因素，为患者制定个体化治疗方案是取得最佳疗效的关键。在诊断为SCLC和治疗的过程中，患者往往伴随着巨大的心理压力，因此，为患者提供心理支持，调整心态可增强患者应对疾病的能力。在整个治疗过程中根据患者具体身体情况制定个体化饮食计划，维持良好的营养状况，对于患者康复至关重要。

SCLC的全程管理在我国还处于初步阶段，目前还没有专门针对SCLC的一套完整的全程随访管理体系，围绕SCLC的全程管理知识存在缺口，诊治中需要多科室的协作。医疗资源的匹配、服务管理的连续性、信息反馈的及时性等使极易复发、治疗效果不佳、需要频繁就诊的SCLC患者的全程管理面临巨大挑战。与此同时SCLC诊疗同质化和规范性稍有欠缺，不同医院甚至不同科室给出的治疗方案差异化较大，区域全程管理中心的上下转诊通道尚未确定，还需要长时间的实践，以便提出更符合SCLC的全程随访管理模式。

随着科技的进步和医疗水平的提高，利用大数据及人工智能，不断优化诊疗流程，提高治疗效果，制定个体化随访方案，进行长期和规律的随访管理，在全程管理的共同努力下，可为更多的SCLC患者带来生命的希望。
